# The Co-occurrence of Chronic Hepatitis B and Fibrosis Is Associated With a Decrease in Hepatic Global DNA Methylation Levels in Patients With Non-alcoholic Fatty Liver Disease

**DOI:** 10.3389/fgene.2021.671552

**Published:** 2021-07-14

**Authors:** FangYuan Li, Qian Ou, ZhiWei Lai, LiuZhen Pu, XingYi Chen, LiRong Wang, LiuQiao Sun, XiaoPing Liang, YaoYao Wang, Hang Xu, Jun Wei, Feng Wu, HuiLian Zhu, LiJun Wang

**Affiliations:** ^1^Department of Nutrition, School of Medicine, Jinan University, Guangzhou, China; ^2^Department of Science and Technology, Guangzhou Customs, Guangzhou, China; ^3^Department of Nutrition, School of Public Health, Sun Yat-sen University, Guangzhou, China

**Keywords:** global DNA methylation, chronic hepatitis B, fibrosis, non-alcoholic fatty-liver disease, inflammation

## Abstract

Global DNA hypomethylation has been reported in patients with chronic hepatitis B (CHB) and non-alcoholic fatty-liver disease (NAFLD). However, the global DNA methylation profile of patients with concurrent NAFLD and CHB (NAFLD + CHB) is still unclear. We aimed to detect the hepatic global DNA methylation levels of NAFLD + CHB patients and assess the associated risk factors. Liver biopsies were collected from 55 NAFLD patients with or without CHB. The histological characteristics of the biopsy were then assessed. Hepatic global DNA methylation levels were quantified by fluorometric method. The hepatic global DNA methylation levels in NAFLD + CHB group were significantly lower than that in NAFLD group. Participants with fibrosis showed lower levels of hepatic global DNA methylation than those without fibrosis. Participants with both CHB and fibrosis had lower levels of hepatic global DNA methylation than those without either CHB or fibrosis. The co-occurrence of CHB and fibrosis was significantly associated with a reduction in global DNA methylation levels compared to the absence of both CHB and fibrosis. Our study suggests that patients with NAFLD + CHB exhibited lower levels of global DNA methylation than patients who had NAFLD alone. The co-occurrence of CHB and liver fibrosis in NAFLD patients was associated with a decrease in global DNA methylation levels.

## Introduction

Non-alcoholic fatty-liver disease (NAFLD) is poised to become a predominant cause of chronic liver disease worldwide (Loomba and Sanyal, 2013). If obesity and diabetes mellitus (DM) stabilize in the future, it is predicted that there will be a modest growth in total NAFLD cases (0–30%) between 2016 and 2030, with the highest growth expected to be seen in China, due to the effects of urbanization (Estes et al., 2018). Chronic hepatitis B (CHB) is a disease caused by hepatitis B virus (HBV) infection and affects more than 257 million individuals worldwide. The rising prevalence of obesity and metabolic syndrome has resulted in an increase in the number of patients with concurrent NAFLD and CHB (NAFLD + CHB).

Both CHB and NAFLD are leading causes of liver-related morbidity and mortality. NAFLD comprises a spectrum of liver diseases that includes simple steatosis, non-alcoholic steatohepatitis, fibrosis, and cirrhosis, and ultimately develops into hepatocellular carcinoma (HCC) (Younossi et al., 2015). CHB contributes substantially to the global disease burden owing to its high prevalence and probability of progression to cirrhosis and HCC (Shen et al., 2012; Lok et al., 2017). Positive hepatitis B core antibody (anti-HBc) have been associated with cirrhosis and possibly HCC in Chinese patients with NAFLD (Chan et al., 2020). In a follow-up study, patients with NAFLD + CHB were found to have a 7.3-fold increased risk of HCC compared to patients with CHB alone (Chan et al., 2017). These studies highlight the challenge of preventing and treating NAFLD + CHB. However, the underlying mechanisms of this compound disease remain unclear.

DNA methylation is the best-known and most studied epigenetic modification, and it refers to heritable changes in gene expression associated with modifications of DNA that are not due to any alteration in the DNA sequence. DNA methylation plays a key role in transcriptional regulation by silencing genes through hypermethylation or activating genes through hypomethylation (Shen et al., 2012; Murphy et al., 2013; Zhang et al., 2013; Zeybel et al., 2016). In addition to gene-specific DNA methylation, the loss of global DNA methylation in sequences that are normally methylated, such as the repetitive sequences *LINE-1* and satellite-2 and inter-spread cytosine-phosphate-guanine (CpG) islands, can lead to chromosomal abnormalities, chromosomal instability, chromosome fragility, and ultimately the development of disease (Amir et al., 2012; Nishida et al., 2013; Patchsung et al., 2018).

DNA methylation accounts for the impacts of environmental factors on liver disease. Marked decrease in the global DNA methylation level was detected in the livers of NAFLD and HCC mice model fed with high fat diet and methyl donor deficient diet (choline, methionine, folic acid, and vitamin B12 deficiency) (Tryndyak et al., 2011; Wang et al., 2014). Similar change also occurs in chemically induced NAFLD and HCC (Chen et al., 2004; Tao et al., 2005; Komatsu et al., 2012). In addition, HBV X protein (HBx) encoding by HBV X gene induces global hypomethylation of satellite-2 repeating sequences (Park et al., 2007). HBx is required for the virus infection and has been shown to induce demethylation of distal regulatory regions to facilitate HCC tumorigenesis (Lee et al., 2014).

Global DNA hypomethylation has also been implicated in HBV exposed HCC patients (Zhang et al., 2013). Furthermore, reduced global DNA methylation of *LINE-1* in white blood cells has been associated with a twofold higher risk of HCC in hepatitis B surface antigen (HBsAg) carriers (Wu et al., 2012). These studies indicate that HBV exposure contributes to a decrease in global DNA methylation and a subsequent increase in the risk of developing HCC. Findings from our laboratory and others have shown that the NAFLD and HCC patients have lower levels of global DNA methylation than corresponding controls (Wu et al., 2012; Nishida et al., 2013; Wang et al., 2014; Lai et al., 2020). However, whether the co-occurrence of CHB with NAFLD further aggravates global DNA hypomethylation in NAFLD patients has not been evaluated.

The histological progression of liver diseases is associated with a decrease in global DNA methylation levels, especially in the case of fibrosis. Studies of animal liver fibrosis models induced by a methionine-choline-deficient diet have reported that the global DNA methylation level in the liver is reduced in these animals (Tryndyak et al., 2011; Page et al., 2016). Mouse models with early stage liver fibrosis also display hypomethylation (Komatsu et al., 2012). In advanced biliary atresia patients, DNA hypomethylation in blood was observed with severe fibrosis compared with mild fibrosis (Udomsinprasert et al., 2016). However, it is not clear whether the superposition of fibrosis on CHB is associated with the decrease in levels of global DNA methylation in NAFLD patients.

Given this uncertainty, we analyzed liver biopsies from NAFLD patients to test whether the co-occurrence of CHB with NAFLD aggravates global DNA hypomethylation, and whether the presence of concurrent CHB and liver fibrosis in NAFLD patients is also associated with a decrease in global DNA methylation levels.

## Materials and Methods

### Human Subjects

The Medical Ethics Committee of the School of Public Health, Sun Yat-sen University (SYSU) approved the study protocol [Project identification code: (2012) No. 17]. Our study protocol conformed to the ethical guidelines of the 1975 Declaration of Helsinki. All subjects signed written informed consent forms prior to the study, which were then collected prior to the day of surgery.

### Study Design

Fifty-five NAFLD patients were recruited from the 157th Hospital in Zhangzhou city, Fujian province in China between June 2012 and June 2013. A percutaneous liver biopsy was obtained using a disposable Menghini needle or an 18-gauge BARD Max-Core Disposable Biopsy Instrument.

Non-alcoholic fatty-liver disease diagnosis was based on the histological evidence for hepatic steatosis via percutaneous liver biopsy (more than 5% of steatosis in the proportion). CHB was defined as having medical records with a positive HBsAg for longer than 6 months. Participants who met the following criteria were excluded from analysis: alcohol consumption (>20 g per day for males, >10 g per day for females); use of antiviral therapy in the 6 months prior to the study period; diagnosis of other viral hepatitis conditions, malignancy, autoimmune liver diseases, or severe hepatic injury or cirrhosis; vitamin use; weight change of more than 2 kg in a single year; and presence of a drug-induced or parental nutrition-induced fatty liver.

### Demographic, Anthropometric, and Biochemical Evaluation

A face-to-face questionnaire was used to assess all participants before performing the liver biopsy. We collected the following information using the questionnaire: socio-demographic characteristics (e.g., age, sex, education level, occupation); lifestyle habits (e.g., alcohol, tobacco, and tea consumption); physical activities; and history of chronic diseases and medication (Chen et al., 2015). Physical activities were expressed as their metabolic equivalents.

When measuring the body weight, height, neck, and waist/hip circumferences, the participants were barefoot and wore light clothing. Body mass index (BMI) was calculated using the formula of weight in kilograms divided by height in meters squared. Systolic and diastolic blood pressure measurements were performed on the right arms of the participants after they had been sitting for at least 10 min.

Fasting serum samples were isolated and stored at −80°C until further analysis. Serum alanine aminotransferase, alanine transaminase, total cholesterol, triglyceride, low-density lipoprotein, high-density lipoprotein, apolipoprotein A, apolipoprotein B, fasting blood glucose, and uric acid concentrations were obtained from patients’ records.

### Histopathological Assessment

Liver samples were stained using both the hematoxylin and eosin and Masson’s trichrome methods. The histological assessment was performed by microscopic examination in accordance with the non-alcoholic steatohepatitis Clinical Research Network Scoring System (Kleiner et al., 2005). Two experienced pathologists, who were blind to the participants’ information, conducted the assessments. The severity of steatosis was graded from 0 to 3 based on the proportion of steatosis: grades 0 (<5%), 1 (5–33%), 2 (33–66%), and 3 (≥66%). The steatosis grade was defined as either mild (grade 1) or moderate (grades 2 and 3) based on the above assessments. Based on the number of inflammatory foci observed per field of view at a magnification of 200×, inflammation was classified as grades 0 (none), 1 (<2 foci/field), 2 (2–4 foci/field), and 3 (≥4 foci/field). The inflammation grade was defined as either mild (grades 0 and 1) or moderate (grades 2 and 3) based on the above assessments. Fibrotic severity was graded from 0 to 4 in accordance with the Brunt classification system: grades 0 (none), 1 (peri-sinusoidal or peri-cellular), 2 (fibrosis in both the peri-sinusoidal sinus and portal vein), 3 (bridging fibrosis without obvious cirrhosis), and 4 (cirrhosis). The total NAFLD activity score (NAS) was calculated as the sum of grades for steatosis, hepatocellular ballooning, and lobular inflammation. The obtained score was then categorized as simple steatosis (SS) (<3), steatohepatitis borderline (NASH-B) (3–4), and non-alcoholic steatohepatitis (NASH) (≥5).

### DNA Methylation Analysis

Genomic DNA was isolated from liver tissue using a TIANamp Genomic DNA Kit (TIANGEN, Beijing, China). The extracted DNA was quantified using a NanoDrop^®^ ND-1000 UV-Vis spectrophotometer (Thermo Scientific, Loughborough, United Kingdom). The global DNA methylation levels in liver tissues were determined using the MethylFlash Methylated DNA Quantification Kit (catalog No. P-103596, fluorometric; EpiGentek Group Inc., New York, NY, United States) according to the manufacturer’s instructions. Each sample was run in duplicate.

### Statistical Analysis

EpiData was used for data input and Statistical Package for Social Sciences (SPSS) v23.0 was used for data analysis. Non-normal variables were presented as medians (interquartile range) and compared using the Mann–Whitney *U* test. Quantitative data were presented as means ± standard deviation (SD) if they were normally distributed. A *t*-test was used to compare the means of anthropometric characteristics, biological characteristics, and hepatic DNA methylation levels, based on the CHB status and histological grade of the study population. A one-way analysis of variance was used to compare the levels of hepatic DNA methylation between groups that were stratified by CHB status and histological characteristics. A chi-square test was used to analyze the distribution of liver histological grades between the NAFLD and NAFLD + CHB groups. The relationships between global DNA methylation level and anthropometric characteristics, biological characteristics, and hepatic histological characteristics were analyzed using univariate and multivariate linear regression. *P* < 0.05 was set as the significance threshold.

## Results

### Baseline Characteristics of the Participants

The anthropometric and biochemical characteristics of the participants, classified based on the presence or absence of CHB, are shown in Table 1. After adjusting for age, sex, and BMI, none of the items were found to be significantly different between the NAFLD and NAFLD + CHB groups. Table 2 shows a comparison of histological assessments between the NAFLD and NAFLD + CHB groups. Fewer patients in the NAFLD + CHB group had moderate steatosis than in the NAFLD group (steatosis grade 2–3). There were no differences in terms of inflammation, fibrosis grade, and NAFLD progression between the two groups. However, after adjusting for anthropometric and biological characteristics, the logistic regression analysis revealed that fibrosis was a risk factor for NAFLD with CHB (OR = 9.723, *P* = 0.037).

**TABLE 1:** Anthropometric and biochemical characteristics of the study population based on the chronic hepatitis B status.

	**NAFLD (*n* = 37)**	**NAFLD + CHB (*n* = 18)**	***P***	*P*_1*_
**Anthropometric characteristic**				
Age, years	34.30 ± 10.84	35.78 ± 9.65	0.625	–
Male, n (%)	30.00 (81.10)	14.00 (77.8)	0.779	–
BMI, kg/m^2^	28.20 ± 3.33	27.35 ± 2.60	0.350	–
Weight, kg	80.86 ± 13.14	76.08 ± 8.18	0.164	0.192
Height, cm	170.00 (166.50, 175.0)	167.50 (162.25, 170.25)	0.102	0.283
Waist circumference, cm	96.63 ± 7.90	94.41 ± 5.52	0.292	0.572
Hip circumference, cm	103.43 ± 6.70	101.07 ± 5.76	0.205	0.423
Waist/hip ratio	0.93 ± 0.48	0.94 ± 0.05	0.942	0.888
Neck circumference, cm	40.01 ± 2.98	40.31 ± 2.70	0.719	0.167
**Biochemical characteristic**				
SBP, mmHg	126.12 ± 11.90	123.67 ± 15.91	0.524	0.209
DBP, mmHg	83.35 (73.00, 90.25)	86.50 (69.00, 90.25)	0.713	0.654
ALT, U/L	67.80 (33.20, 100.50)	44.75 (25.28, 76.60)	0.274	0.898
AST, U/L	31.90 (25.00, 47.35)	24.95 (19.95, 32.55)	0.136	0.893
ALT/AST	0.51 (0.41, 0.72)	0.65 (0.48, 0.70)	0.216	0.554
TG, mmol/L	1.81 (1.31, 2.65)	1.64 (1.02, 2.21)	0.524	0.630
TC, mmol/L	5.05 (4.51, 5.76)	4.88 (4.48, 5.52)	0.760	0.602
HDL, mmol/L	1.20 (1.05, 1.30)	1.15 (0.97, 1.36)	0.740	0.596
LDL, mmol/L	2.98 (2.52, 3.44)	2.82 (2.56, 3.47)	0.865	0.838
APOA, g/L	1.44 (1.26, 1.56)	1.36 (1.29, 1.54)	0.879	0.786
APOB, g/L	1.03 (0.86, 1.14)	1.06 (0.92, 1.28)	0.490	0.873
Uric acid, μM	418.61 ± 108.93	361.88 ± 105.75	0.073	0.104
Glucose, mmol/L	5.05 (4.73, 5.98)	5.08 (4.73, 6.34)	0.993	0.539

Data are expressed as means ± standard deviation (SD) or median (interquartile range) based on the variable distribution. *P_1_: *P* value was adjusted for age, sex, and body mass index. ALT, alanine aminotransferase; APOA, apolipoprotein A; APOB, apolipoprotein B; AST, aspartate aminotransferase; BMI, Body mass index; CHB, chronic hepatitis B; DBP, diastolic blood pressure; HDL, high-density lipoprotein; LDL, low-density lipoprotein; NAFLD, non-alcoholic fatty liver disease; NAFLD + CHB, concurrent non-alcoholic fatty liver disease and chronic hepatitis B; SBP, systolic Blood Pressure; TC, total cholesterol; TG, triglyceride.

**TABLE 2:** Liver pathology of the study population based on the disease status, n (%).

**Factor**	**NAFLD (*n* = 37)**	**NAFLD + CHB (*n* = 18)**	P*
Steatosis grade			**0.016**
1 (mild)	16 (43.2)	14 (77.8)	
2–3 (moderate)	21 (56.8)	4 (22.2)	
Inflammation grade			0.196
0–1 (mild)	27 (73.0)	10 (55.6)	
2–3 (moderate)	10 (27.0)	8 (44.4)	
Fibrosis grade			0.141
0	18 (48.6)	5 (27.8)	
1–3	19 (51.4)	13 (72.2)	
NAFLD progression			0.282
SS	11 (29.7)	8 (44.4)	
NASH-B	26 (70.3)	10 (55.6)	

*P values in bold indicate a significant difference. CHB, chronic hepatitis B; NAFLD, non-alcoholic fatty liver disease; NAFLD + CHB, concurrent non-alcoholic fatty liver disease and chronic hepatitis B. NAFLD progression based on the NAFLD activity score (NAS), graded as simple steatosis (SS) and steatohepatitis borderline (NASH-B).

### Hepatic Global DNA Methylation Levels Based on CHB Status and Histological Characteristics

**FIGURE 1:**
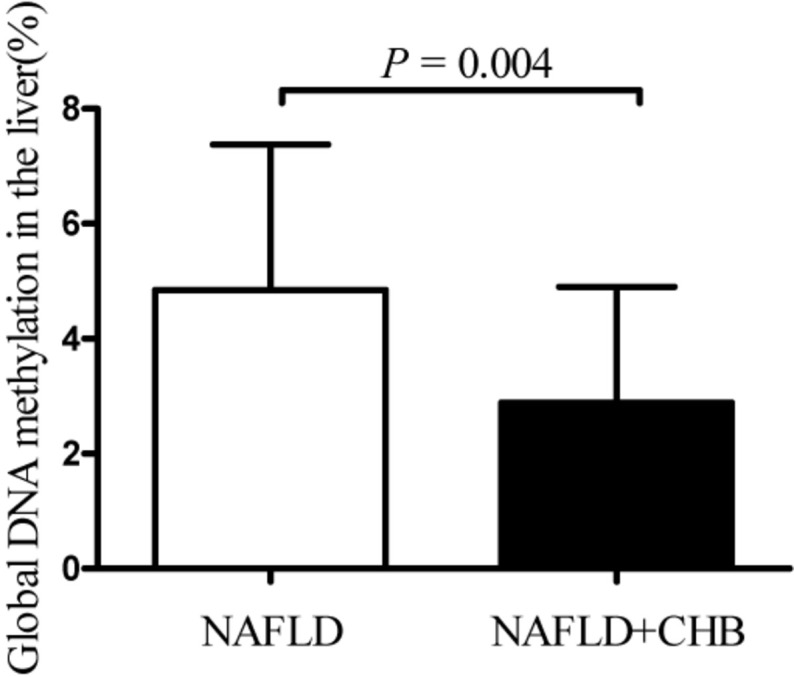
Hepatic global DNA methylation levels based on chronic hepatitis B status. Data are expressed as means with standard deviation (SD). CHB, chronic hepatitis B; NAFLD, non-alcoholic fatty liver disease; NAFLD + CHB, concurrent non-alcoholic fatty liver disease and chronic hepatitis B. *P-*value was adjusted for age, sex, and body mass index.

Compared to NAFLD subjects, the global DNA methylation levels in the livers of NAFLD + CHB patients were significantly reduced, by 34.85% (NAFLD vs. NAFLD + CHB: 4.85 vs. 3.16%, *P* = 0.017). After adjusting for age, sex, and BMI, the difference remained significant (*P* = 0.004) (Figure 1). The differences in global DNA methylation levels for each histological grade are shown in Table 3. Participants with fibrosis had significantly lower levels of global DNA methylation than those without fibrosis (*P* = 0.026). With respect to steatosis grade, inflammation grade, and NAFLD progression, the global DNA methylation levels were not significantly different between the groups.

**TABLE 3:** Global DNA methylation levels for each histological grade across the study population.

**Factor**	**Global DNA methylation (%)**	***P*^∗^**
Steatosis grade		0.155
1 (mild)	3.85 ± 2.16	
2-3 (moderate)	4.83 ± 2.91	
Inflammation grade		0.111
0-1 (mild)	4.68 ± 2.72	
2-3 (moderate)	3.51 ± 2.03	
Fibrosis grade		**0.026**
0	5.19 ± 2.59	
1-3	3.65 ± 2.36	
NAFLD progression^#^		0.943
SS	4.33 ± 2.17	
NASH-B	4.28 ± 2.76	

*Data are expressed as means ± standard deviation (SD). *^∗^P*-values in bold indicate a significant difference. ^#^NAFLD progression based on the NAFLD activity score (NAS), graded as simple steatosis (SS) and steatohepatitis borderline (NASH-B).*

### Hepatic Global DNA Methylation Levels in the NAFLD and NAFLD+CHB Groups as Stratified by Histological Severity

**FIGURE 2:**
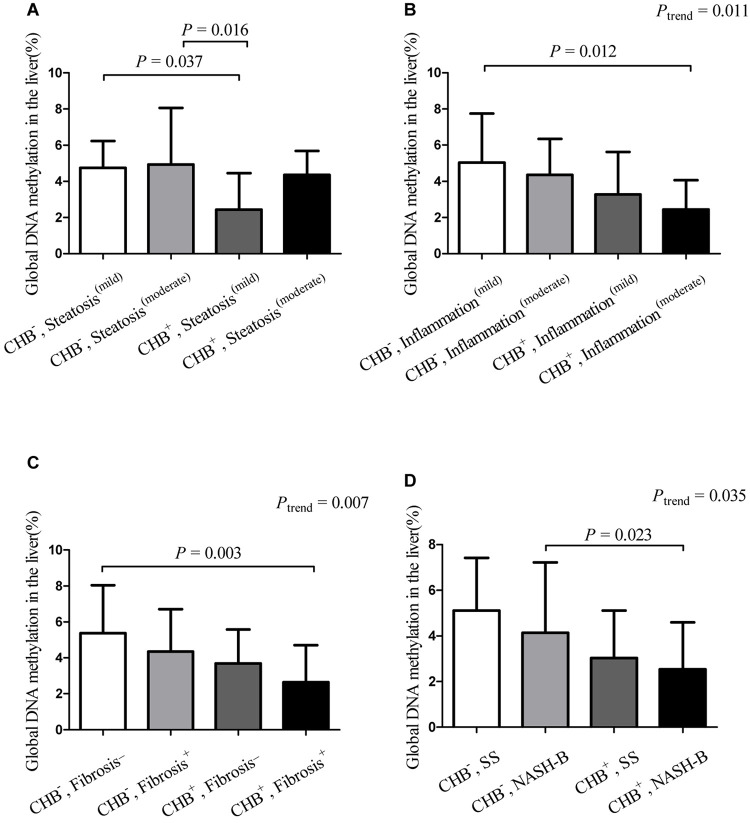
Hepatic global DNA methylation levels in groups stratified by chronic hepatitis B status and histological characteristics*^∗^*. Data are expressed as means with standard deviation (SD). *^∗^*Hepatic global DNA methylation levels in groups, which were stratified by chronic hepatitis B status and steatosis grade **(A)**, inflammation grade **(B)**, the presence or absence of fibrosis **(C)**, NAFLD activity score **(D)**. CHB^–^, non-alcoholic fatty liver disease without chronic hepatitis B; CHB^+^, concurrent non-alcoholic fatty liver disease and chronic hepatitis B; Fibrosis^–^, without fibrosis; Fibrosis^+^, concurrent with fibrosis; Inflammation^(mild)^, mild inflammation (grades 0 and 1); Inflammation^(moderate)^, moderate inflammation (grades 2 and 3); NASH-B, steatohepatitis borderline; SS, simple steatosis; Steatosis^(mild)^, mild steatosis (grade 1); Steatosis^(moderate)^, moderate steatosis (grades 2 and 3).

We further compared the differences in global DNA methylation levels between the NAFLD + CHB and NAFLD groups, which were stratified by histological grade (Figure 2). This revealed that the presence of CHB significantly decreased the global DNA methylation levels in subjects with mild steatosis (with both CHB and mild steatosis vs. without CHB and with mild steatosis: 2.82 vs. 4.75%, *P* = 0.037) (Figure 2A). Compared to patients without CHB and with mild inflammation, those with both CHB and concomitant moderate inflammation had lower levels of global DNA methylation (without CHB and with mild inflammation vs. with both CHB and moderate inflammation: 5.03 vs. 2.45%, *P* = 0.012) (Figure 2B). Compared to those without CHB and fibrosis, those with both CHB and fibrosis had lower levels of global DNA methylation (without CHB or fibrosis vs. with both CHB and fibrosis: 5.38 vs. 2.64%, *P* = 0.003) (Figure 2C). The presence of CHB significantly decreased the global DNA methylation levels in subjects with borderline steatohepatitis (with both CHB and borderline steatohepatitis vs. without CHB and with borderline steatohepatitis: 2.72 vs. 4.88%, *P* = 0.023) (Figure 2D). The global DNA methylation levels showed a decreasing trend in the presence of co-occurrent CHB and inflammation progression (*P*_trend_ = 0.011), co-occurrent CHB and presence of fibrosis (*P*_trend_ = 0.007), and co-occurrent CHB and NAFLD progression (*P*_trend_ = 0.035).

### The Correlation of Global DNA Methylation Levels With Anthropometric, Biochemical, and Histological Characteristics

Upon conducting univariate regression analysis, we found that BMI was negatively correlated with global genomic methylation levels, although anthropometric and biochemical characteristics did not share this association (Supplementary Table 1). Table 4 shows the results of the multivariate analysis after adjusting for anthropometric and biochemical characteristics. In subjects with mild steatosis, the presence of CHB was negatively associated with global DNA methylation levels when compared to the absence of CHB (reference). The presence of CHB and moderate inflammation was negatively associated with global DNA methylation levels when compared to the group that had no CHB but had mild inflammation (reference). The presence of both CHB and fibrosis was negatively associated with global DNA methylation levels when compared to the absence of both CHB and fibrosis (reference).

**TABLE 4:** Multivariable analysis of global DNA methylation and histological variables in patients after adjusting for anthropometric and biochemical variables.

**Variable**	**Multivariable analysis**	

	**Global DNA methylation**	***P*^∗^**
**CHB and Steatosis**		
CHB^–^, Steatosis^(mild)^	Reference	–
CHB^–^, Steatosis^(moderate)^	−0.01 (−1.81, 1.75)	0.974
CHB^+^, Steatosis^(mild)^	−0.56 (−5.14, −1.39)	**0.001**
CHB^+^, Steatosis^(moderate)^	0.08 (−2.08, 3.61)	0.589
**CHB and Inflammation**		
CHB^–^, Inflammation^(mild)^	Reference	–
CHB^–^, Inflammation^(moderate)^	−0.00 (−2.09, 2.07)	0.992
CHB^+^, Inflammation^(mild)^	−0.27 (−3.63, 0.12)	0.066
CHB^+^, Inflammation^(moderate)^	−0.38 (−4.88, −0.53)	**0.016**
**CHB and Fibrosis**		
CHB^–^, Fibrosis^–^	Reference	–
CHB^–^, Fibrosis^+^	−0.29 (−3.41, 0.29)	0.094
CHB^+^, Fibrosis^–^	−0.21 (−4.53, 0.86)	0.175
CHB^+^, Fibrosis^+^	−0.55 (−5.15, −1.40)	**0.001**
**CHB and NAFLD progression**		
CHB^–^, SS	Reference	–
CHB^–^, NASH-B	0.02 (−1.91, 2.15)	0.907
CHB^+^, SS	−0.28 (−4.40, 0.46)	0.108
CHB^+^, NASH-B	−0.32 (−4.54, 0.31)	0.085

*Data are expressed as beta (95% CI). *^∗^P*-values in bold indicate a significant difference. CHB^–^, non-alcoholic fatty liver disease without chronic hepatitis B; CHB^+^, concurrent non-alcoholic fatty liver disease and chronic hepatitis B; Fibrosis^–^, without fibrosis; Fibrosis^+^, concurrent with fibrosis; Inflammation^(mild)^, mild inflammation (grades 0 and 1); Inflammation^(moderate)^, moderate inflammation (grades 2 and 3); NASH-B, steatohepatitis borderline; SS, simple steatosis; Steatosis^(mild)^, mild steatosis (grade 1); Steatosis^(moderate)^, moderate steatosis (grades 2 and 3).*

## Discussion

This is the first study to report that patients with concurrent NAFLD and CHB show significantly lower levels of hepatic global DNA methylation than patients with NAFLD alone. The presence of both CHB and fibrosis was associated with decreased hepatic global DNA methylation levels, compared to those of patients who had neither of these conditions.

DNA methylation is one kind of epigenetic modifications that connect environment and disease. Feeding a methyl donor deficient diet caused loss of global DNA methylation in the livers of rodents and induced hepatic fibrosis and HCC (Tryndyak et al., 2011). DNA hypomethylation was also found in the livers of rodents with fatty liver induce by high fat diet and alcohol (Lu et al., 2000; Wang et al., 2014). On the contrary, methyl donor supplementation could restore the global DNA methylation level and reversed liver injury (Medici et al., 2013). In addition to diet, global DNA hypomethylation can be induced by many carcinogenic chemicals in the cells or livers such as arsenic, chromium (Takiguchi et al., 2003; Chen et al., 2004). Hepatitis infection is another risk factor to induce DNA hypomethylation. *In vivo* studies on human hepatocyte chimeric mouse models have shown that the levels of *LINE-1* methylation in long-term HBV- and HCV-infected mice are lower than those in uninfected control mice (Okamoto et al., 2014). Another *in vivo* study demonstrated that the HBV X protein induces global hypomethylation of satellite-2 repeating sequences (Park et al., 2007). Furthermore, global DNA hypomethylation has been implicated in HBV-exposed HCC patients (Zhang et al., 2013). Similarly, as we observed that the levels of global DNA methylation in NAFLD + CHB patients were lower than those in NAFLD patients.

However, the mechanisms for the decrease in global DNA methylation levels upon the co-occurrence of CHB is not very clear. DNA methylation is a process that transforms 5′-cytosine into 5′-methylcytosine with the adding methyl group from the universal methyl donor-S-adenosylmethionine (SAM). This reaction is catalyzed by a family of enzymes known as DNA methyltransferases (DNMTs). Several possibilities that may contribute to the development of DNA hypomethylation have been proposed. One underlying mechanism is the reduction of methylation capacity because of intracellular depletion of SAM. SAM is an essential and critical methyl donor for cellular transmethylation reactions including DNA, RNA, and histone methylation (Ouyang et al., 2020). Diet low in source of methyl donors, high in fat can lead to global DNA hypomethylation in the livers by impairing synthesis of SAM, which could be reversed by methyl donor supplementation (Medici et al., 2013; Wang et al., 2014; Bakir et al., 2019). However, the change of one-carbon metabolism in HBV is still unclear. The other mechanism is the changes in DNMTs expression and/or activity. HBV-induced aberrant global DNA hypomethylation is associated with the reduced expression of genes *DNMT1* and *DNMT3b* (Park et al., 2007; Huang et al., 2010). Aside from regulating the transcription of *DNMT* genes, HBV also affects the capacity of these genes to bind their corresponding regulatory elements. A study using chromatin immunoprecipitation assays showed that HBV partially or completely abrogates *DNMT3a* binding to target gene promoters, contaminant with a decreased DNA methylation in regulatory elements (Zheng et al., 2009). Therefore, the global DNA hypomethylation of NAFLD patients with CHB may be associated with downregulated expression and activity of DNMTs. The DNA integrity is another critical factor that affects the normal status of DNA methylation (Pogribny and Beland, 2009). Diminished methylation capacity of DNA methyltransferases, leading to DNA hypomethylation can be induced by the presence of unrepaired lesions in DNA, such as 8-oxoguanine and 5-hydroxymethylcytosine (Pogribny and Beland, 2009). It has been reported that diet-induced NAFLD is associated with revisable 5-hydroxymethylcytosine change in the liver (Lyall et al., 2020). While the effect of HBV on genome integrity has not been reported yet.

Our study found that the presence of both CHB and fibrosis in NAFLD patients significantly reduced global DNA methylation levels compared to those in the absence of both conditions. After adjusting for anthropometric and biochemical characteristics, the presence of CHB and fibrosis was negatively associated with global DNA methylation levels in the liver. Hepatic global DNA methylation level is reduced in animal liver fibrosis models induced by a methionine-choline-deficient diet and intraperitoneal injection of CCl_4_ (Tryndyak et al., 2011; Komatsu et al., 2012; Page et al., 2016). In addition, DNA hypomethylation has been observed in the blood of advanced biliary atresia patients with severe fibrosis, compared to those with mild fibrosis (Udomsinprasert et al., 2016). The above data from our study and others indicated that DNA hypomethylation may be involved in the pathological progress of liver diseases. While there is no consensus on how DNA hypomethylation promotes the development of disease. Generally, molecular mechanisms of global hypomethylation on adverse outcome may be attributed to the dysregulation of chromosomal abnormalities, and genomic instability. First, global DNA hypomethylation may lead to chromosomal abnormalities. Demethylation of repetitive sequences located at centromeric, pericentromeric, and subtelomeric chromosomal regions may cause the induction of chromosomal abnormalities (Pogribny and Beland, 2009). It has been reported that DNA hypomethylation in HCCs is clearly associated with the amount of chromosomal alterations (Nishida et al., 2013). Second, global DNA hypomethylation may promote chromosomal instability (CIN), which has been proved to be mediated by DNA damage. The increase of DNA strand breaks precedes DNA hypomethylation (James et al., 2003), and DNA damage is a precursor of mutation (Patchsung et al., 2018) and can lead to related pathological findings. In addition, global DNA hypomethylation can also lead to chromosome fragility, which in turn leads to CIN in HCC (Nishida et al., 2013). For example, global DNA hypomethylation promotes early liver tumor formation by leading to aneuploidy, chromosome translocation and copy number changes in HCC mouse models, human hepatoma cell lines (Yamada et al., 2005; Tryndyak et al., 2011; Komatsu et al., 2012; Udomsinprasert et al., 2016).

There are several limitations to the present study. First, the number of subjects was relatively small, leading to lack of power to determine the statistical differences in global DNA methylation levels between different groups. Second, the data were obtained from cross-sectional analyses, and we therefore could not detect the causal relationship between global DNA methylation and HBV infection or pathological changes. Third, this is an association study, so we cannot sum up the causes, mechanisms and consequences by itself. Whether or not there is an association between changes in DNA-methylation and gene expression remains unclear and should be investigated in following studies.

Despite the limitations of this study, we have assessed the level of global DNA methylation in patients who are affected by both NAFLD and CHB, which are rarely involved in previous studies. We also evaluated the association between global DNA hypomethylation and liver pathology. As the overexpression of oncogenes caused by DNA hypomethylation may play an important role in tumorigenesis (Good et al., 2018), global DNA methylation could be used to characterize the epigenetic characteristics of these patients. Our data also provide a theoretical basis for further investigations into the epigenetics of chronic liver disease.

In conclusion, our study shows that patients with concurrent NAFLD and CHB exhibited lower levels of global DNA methylation than patients with NAFLD alone. The co-occurrence of CHB and liver fibrosis in NAFLD patients was associated with a decrease in global DNA methylation levels. Our findings provide new insights into the epigenetic events underpinning NAFLD + CHB and may provide the basis for new research into specific epigenetic modifications mediated by virus.

## Data Availability Statement

The raw data supporting the conclusions of this article will be made available by the authors, without undue reservation.

## Ethics Statement

The studies involving human participants were reviewed and approved by the Medical Ethics Committee of the School of Public Health, Sun Yat-sen University (SYSU) approved the study protocol (Project identification code: [2012] No. 17). The patients/participants provided their written informed consent to participate in this study. Written informed consent was obtained from the individual(s) for the publication of any potentially identifiable images or data included in this article.

## Author Contributions

FYL and LJW designed the research study, analyzed the data, and wrote the manuscript. QO, ZWL, LZP, XYC, LRW, LQS, XPL, YYW, HX, JW, and FW performed the research and collected liver biopsy and other biological samples. HLZ was acting as the submission’s guarantor and revised the manuscript. All authors approved the final version of the article, including the authorship list.

## Conflict of Interest

The authors declare that the research was conducted in the absence of any commercial or financial relationships that could be construed as a potential conflict of interest.

## Supplementary Material

Supplementary Table 1 Univariate analysis of global DNA methylation and anthropometric, biochemical characteristics and histological variables in patients.

## Abbreviations

ALT: alanine aminotransferase
APOA: apolipoprotein A
APOB: apolipoprotein B
AST: aspartate aminotransferase
BMI: Body mass index
CHB: chronic hepatitis B
CIN: chromosomal instability
CpG: cytosine-phosphate-guanine
DBP: diastolic blood pressure
DM: diabetes mellitus
*DNMTs*: DNA methyltransferases
HBsAg: hepatitis B virus surface antigen
HBV: hepatitis B virus
HBx: HBV X protein
HCC: hepatocellular carcinoma
HDL: high-density lipoprotein
LDL: low-density lipoprotein
MET: metabolic equivalent
NAFLD: non-alcoholic fatty liver disease
NAFLD + CHB: patients with concurrent NAFLD and CHB
NAS: NAFLD activity score
NASH: non-alcoholic steatohepatitis
NASH-B: steatohepatitis borderline
SAM: S-adenosylmethionine
SS: simple steatosis
SBP: systolic Blood Pressure
TC: total cholesterol
TG: triglyceride.
