# Supplementary material for: The Co-occurrence of Chronic Hepatitis B and Fibrosis Is Associated With a Decrease in Hepatic Global DNA Methylation Levels in Patients With Non-alcoholic Fatty Liver Disease
Source: Front Genet. 2021 Jul 14;12:671552. doi: 10.3389/fgene.2021.671552 (PMC8318039; doi:10.3389/fgene.2021.671552)
Supplement: Supplementary Table 1 — Univariate analysis of global DNA methylation and anthropometric, biochemical characteristics and histological variables in patients. [file Table_1.docx]

**Supplemental Table**

**Supplementary Table 1.** Univariate analysis of global DNA methylation and anthropometric, biochemical characteristics and histological variables in patients.

| Variable | UnivariableAnalysis | |
| --- | --- | --- |
|  | GlobalDNAmethylation | *P^*^* |
| **Anthropometriccharacteristic** |  |  |
| Age | -0.04(-0.11, 0.03) | 0.240 |
| Gender | -1.27(-2.98, 0.44) | 0.142 |
| BMI | -0.23(-0.44, 0.01) | **0.042** |
| Waistcircumference | -0.05(-0.14, 0.05) | 0.350 |
| Hipcircumference | -0.05(-0.16, 0.06) | 0.342 |
| Waist/hipratio | -1.77(-16.27, 12.74) | 0.808 |
| Neckcircumference | -0.00(-0.25, 0.24) | 0.982 |
| **Biochemicalcharacteristic** |  |  |
| SBP | 0.00(-0.05, 0.06) | 0.920 |
| DBP | 0.04(-0.02, 0.11) | 0.180 |
| ALT | -0.00(-0.01, 0.00) | 0.319 |
| AST | -0.01(-0.03, 0.01) | 0.280 |
| ALT/AST | 0.44(-2.29, 3.17) | 0.746 |
| TG | 0.08(-0.61, 0.77) | 0.815 |
| TC | 0.16(-0.45, 0.77) | 0.592 |
| HDL | -0.60(-3.29, 2.08) | 0.655 |
| LDL | -0.07(-0.96, 0.83) | 0.885 |
| APOA | -2.35(-4.78, 0.09) | 0.058 |
| APOB | -0.51(-2.72, 1.70) | 0.646 |
| Glucose | -0.01(-0.24, 0.23) | 0.969 |
| **CHB and Steatosis** |  |  |
| CHB^-^, Steatosis^(mild)^ | 0.64(-0.89, 2.16) | 0.406 |
| CHB^-^, Steatosis^(moderate)^ | 1.02(-0.39, 2.43) | 0.152 |
| CHB^+^, Steatosis^(mild)^ | -1.98(-3.49, -0.48) | **0.011** |
| CHB^+^, Steatosis^(moderate)^ | 0.06(-2.62, 2.74) | 0.964 |
| **CHBandInflammation** |  |  |
| CHB^-^, Inflammation^(mild)^ | 1.45(0.11, 2.78) | **0.034** |
| CHB^-^, Inflammation^(moderate)^ | 0.07(-1.74, 1.88) | 0.938 |
| CHB^+^, Inflammation^(mild)^ | -0.70(-2.49, 1.10) | 0.440 |
| CHB^+^, Inflammation^(moderate)^ | -2.16(-4.05, -0.28) | **0.025** |
| **CHBandFibrosis** |  |  |
| CHB^-^, Fibrosis^-^ | 1.61(0.20, 3.03) | **0.027** |
| CHB^-^, Fibrosis^+^ | 0.08(-1.39, 1.54) | 0.918 |
| CHB^+^, Fibrosis^-^ | 0.22(-2.20, 2.65) | 0.855 |
| CHB^+^, Fibrosis^+^ | -2.16(-3.69, -0.64) | **0.006** |
| **CHBandNAFLD progression** |  |  |
| CHB^-^, SS | 0.61(-1.13, 2.34) | 0.487 |
| CHB^-^, NASH-B | 1.11(-0.26, 2.47) | 0.110 |
| CHB^+^, SS | -0.68(-2.65, 1.28) | 0.489 |
| CHB^+^, NASH-B | -1.93(-3.66, -0.20) | **0.029** |

Dataareexpressed as beta (95% CI).*^*^P* values in bold indicate a significant difference. ALT, alanine aminotransferase; APOA, apolipoprotein A; APOB, apolipoprotein B; AST, aspartate aminotransferase; BMI, Body mass index; CHB, chronic hepatitis B; CHB^-^, non-alcoholic fatty liver disease without chronic hepatitis B; CHB^+^, concurrent non-alcoholic fatty liver disease and chronic hepatitis B; DBP, diastolic blood pressure; Fibrosis^-^, without fibrosis; Fibrosis^+^, concurrent with fibrosis; HDL, high-density lipoprotein; Inflammation^(mild)^, mild inflammation (grades 0 and 1); Inflammation^(moderate)^, moderate inflammation (grades 2 and 3); LDL, low-density lipoprotein; NAFLD,non-alcoholicfattyliverdisease; NASH-B, steatohepatitis borderline. SBP, systolic Blood Pressure; SS, simple steatosis;Steatosis^(mild)^, mild steatosis (grade 1); Steatosis^(moderate)^, moderate steatosis (grades 2 and 3); TC, total cholesterol; TG, triglyceride.
